# RBM45 homo-oligomerization mediates association with ALS-linked proteins and stress granules

**DOI:** 10.1038/srep14262

**Published:** 2015-09-22

**Authors:** Yang Li, Mahlon Collins, Rachel Geiser, Nadine Bakkar, David Riascos, Robert Bowser

**Affiliations:** 1 Divisions of Neurology and Neurobiology, Barrow Neurological Institute, St. Joseph’s Hospital and Medical Center, Phoenix, Arizona 85013, USA; 2 University of Pittsburgh School of Medicine, Pittsburgh, Pennsylvania 15261, USA

## Abstract

The aggregation of RNA-binding proteins is a pathological hallmark of amyotrophic lateral sclerosis (ALS) and frontotemporal lobar degeneration (FTLD). RBM45 is an RNA-binding protein that forms cytoplasmic inclusions in neurons and glia in ALS and FTLD. To explore the role of RBM45 in ALS and FTLD, we examined the contribution of the protein’s domains to its function, subcellular localization, and interaction with itself and ALS-linked proteins. We find that RBM45 forms homo-oligomers and physically associates with the ALS-linked proteins TDP-43 and FUS in the nucleus. Nuclear localization of RBM45 is mediated by a bipartite nuclear-localization sequence (NLS) located at the C-terminus. RBM45 mutants that lack a functional NLS accumulate in the cytoplasm and form TDP-43 positive stress granules. Moreover, we identify a novel structural element, termed the homo-oligomer assembly (HOA) domain, that is highly conserved across species and promote homo-oligomerization of RBM45. RBM45 mutants that fail to form homo-oligomers exhibit significantly reduced association with ALS-linked proteins and inclusion into stress granules. These results show that RMB45 may function as a homo-oligomer and that its oligomerization contributes to ALS/FTLD RNA-binding protein aggregation.

Amyotrophic lateral sclerosis (ALS) is a progressive, fatal neurodegenerative disorder resulting from the death of upper and lower motor neurons. Approximately 90% of all ALS cases are considered sporadic and of unknown etiology. Multiple cellular mechanisms have been proposed to contribute to motor neuron degeneration in sporadic ALS, including altered RNA processing, protein aggregation, mitochondrial defects and oxidative stress^1^. These mechanisms, in particular altered RNA processing and protein aggregation, are pathological phenomena also observed in frontotemporal lobar degeneration (FTLD), which is a progressive, neurodegenerative disease characterized pathologically by neurodegeneration and atrophy of the frontal and temporal cortical lobes of the brain^2^. Neuronal and glial inclusions in the cells from patients with these disorders frequently contain RNA-binding proteins, including TDP-43 (TAR DNA Binding Protein) and FUS (Fused in Sarcoma). Moreover, mutations in TDP-43 and FUS genes are associated with rare familial forms of ALS and FTLD^3^,^4^,^5^,^6^,^7^. Collectively, these observations suggest common mechanisms of neurodegeneration in ALS and FTLD related to the aggregation and loss-of-function of RNA-binding proteins.

Studies of the normal and pathological functions of RNA-binding proteins such as TDP-43 and FUS have provided insight into the role of these proteins in ALS and FTLD. TDP-43 and FUS are multi-functional RNA-binding proteins that contribute extensively to the regulation of gene expression. Both proteins influence pre-mRNA splicing, participate in RNA transport, and modulate RNA stability^8^. In response to a variety of stressors such as heat shock or oxidative insult, TDP-43 and FUS translocate from the nucleus and associate with cytoplasmic stress granules that are dense aggregations of protein-RNA complexes^9^,^10^,^11^. TDP-43 recruited to stress granules under conditions of chronic stress is capable of forming insoluble protein aggregates, even when other components of the stress granules have dissociated from the complex^12^. Protein constituents of stress granules, such as TIA-1, have also been found in cytoplasmic inclusions in motor neurons in ALS^10^. These observations suggest that stress granules represent sites of recruitment of disease-associated RNA-binding proteins that can subsequently lead to pathologic protein aggregation.

Structure-function studies of TDP-43 and FUS have provided considerable insight into the mechanisms by which these and other RNA-binding proteins exert their normal functions, associate with stress granules, and aggregate into inclusion bodies in disorders such as FTLD and ALS. Both TDP-43 and FUS contain RNA-recognition motifs (RRMs) that are necessary for the nucleic acid binding functions of the protein^8^. Each protein also contains a nuclear localization sequence (NLS) that directs the subcellular localization of the protein to the nucleus under normal conditions^13^,^14^. Mutation of the NLS in either protein leads to cytoplasmic retention and inclusion formation in cultured cells^15^,^16^. Each protein also contains a glycine-rich domain that promotes protein aggregation^17^ and their recruitment to stress granules^18^, and is the hot spot for many mutations identified in the *TDP-43* and *FUS* genes of patients with familial forms of ALS and FTLD^19^. Collectively, these results demonstrate how specific domains in RNA-binding proteins contribute to the function and pathology of these proteins and provide a framework for the study of other disease-associated RNA-binding proteins.

One such protein is RBM45, an RNA-binding protein we found in inclusions in the cells of patients with ALS and FTLD. A proteomic analysis of cerebrospinal fluid (CSF) from sporadic ALS and healthy control subjects identified increased levels of RBM45 in the CSF of sporadic ALS patients^20^. RBM45 is expressed predominantly in the nervous system in a developmentally regulated manner^21^. In neurons and glia in a majority of ALS and FTLD patients, RBM45 was also found in intracellular inclusions. Many RBM45 cytoplasmic inclusions contain TDP-43 and are marked with ubiquitin. Similar to TDP-43 and FUS, RBM45 contains RRMs but lacks the glycine-rich domain present in both TDP-43 and FUS. Moreover, RBM45 was found to interact with the C-terminal fragment of TDP-43 in a yeast two-hybrid screen^22^. These results suggest that RBM45 inclusions are a common pathologic phenomenon in ALS and FTLD and that toxic gain or loss of normal RBM45 function may contribute to the pathogenesis of these disorders. At present, little is known about the biologic functions of RBM45 and how it contributes to the progression of neurodegenerative diseases.

In this study, we identify structural determinants of RBM45 for subcellular distribution and protein-protein interactions. Our results show that RBM45 localizes predominately to the nucleus via a bipartite nuclear-localization sequence located at the C-terminal region. In addition, we have identified a novel and evolutionary conserved structural element located within the linker region between RRM2 and RRM3 that is essential for self-association and oligomerization of RBM45. Importantly, the oligomerization of RBM45 mediates its association with TDP-43 as well as inclusion within stress granules.

## Results

### RBM45 localizes predominately in the nucleus

**Figure 1:**
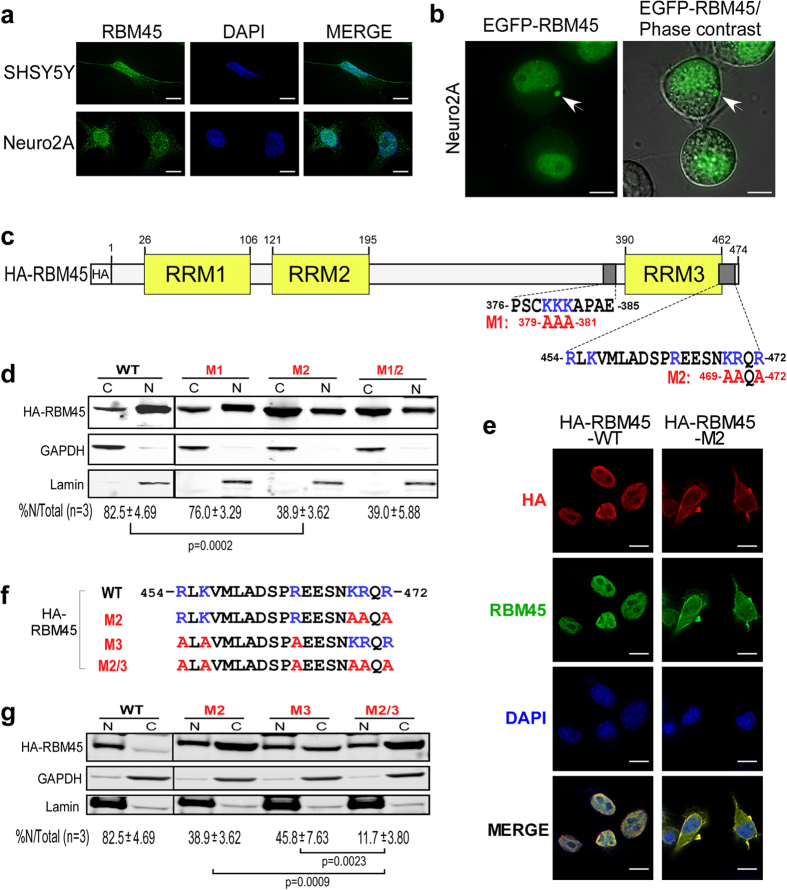
Nuclear localization of RBM45. (**a**) Immunofluorescence of endogenous RBM45 in SHSY5Y and Neuro2A cells that were stained with RBM45 C-terminal antibody (epitope residues 460–474). DAPI was used as the nuclear stain. Scale bar: 10 μm. (**b**) Overexpressed EGFP-RBM45 forms large cytoplasmic granules in Neuro2A cells. Images were taken with living cells grown on glass-bottom plates. Phase contrast is used to delineate cell shape. The cytoplasmic granules are indicated with white arrows. Scale bar: 10 μm. (**c**) Domain structure of RBM45. The RRM domains are shown in yellow. The two predicted NLS are shown as dark grey boxes with their sequences indicated below (residues 376–385, and residues 454–472). The basic residues are highlighted in blue. The NLS mutants M1 and M2 with mutated amino acid residues highlighted in red are shown below the wild-type sequences. (**d**) Subcellular fractionation and immunoblot of the HA-tagged wild-type, M1 mutant, M2 mutant and the double-mutant M1/2 of RBM45. HA-RBM45 constructs were transfected into Neuro2A cells and biochemical fractionation was performed 48 hr post-transfection. Equal proportions of nuclear extract and cytoplasmic extract were immunoblotted with HA (HA-RBM45 constructs), GAPDH (cytoplasmic marker) and Lamin A/C (nuclear marker) antibodies. C = Cytoplasmic fraction, N = Nuclear fraction. The average percentage of nuclear RBM45 in total RBM45 from three experiments and standard deviation are indicated below the blot. The *p*-value was calculated by two-tailed paired t-test. (**e**) Immunofluorescence showing the subcellular localization of the HA-RBM45 wild-type and the M2 mutant in Neuro2A cells. Ectopic HA-RBM45 was stained with mouse HA antibody (red). Total RBM45 was stained with RBM45 C-terminal antibody (green). Nuclei were stained with DAPI. Scale bar: 10 μm. (**f**) Sequence of the bipartite NLS and its mutants. The basic residues are shown in blue and the mutated residues are highlighted in red. M2 mutant contains mutations at the C-end, M3 mutant contains mutations at the N-end, and the double mutant M2/3 contains both mutations at each end. (**g**) Subcellular fractionation and immunoblot of HA-tagged RBM45 wild-type and NLS mutants from **f**. Transfection, fractionation, immunoblot and statistics were performed as described in **d**.

RBM45 was previously detected in cytoplasmic inclusions of neurons and glia in ALS and FTLD patient tissue^20^. To understand the subcellular distribution of RBM45 in neuronal cell types, we performed immunofluorescence staining to detect endogenous RBM45 using an antibody against a C-terminal epitope (residues 460–474) within RBM45 (Supplementary Fig. S1a). Our results showed that endogenous RBM45 in human SHSY5Y and mouse Neuro2A neuroblastoma cells are located predominantly in the nucleus (Fig. 1a). Subcellular fractionation analysis in multiple cell lines including SHSY5Y, Neuro2A and HEK293 also confirmed nuclear localization of endogenous RBM45 (Supplementary Fig. S1b). To visualize the subcellular distribution of RBM45 in live cells, we overexpressed a recombinant EGFP-RBM45 fusion protein in Neuro2A cells. The EGFP-RBM45 fusion protein, similar to the endogenous protein, localized predominately to the nucleus and surprisingly, approximately 10% of the transfected cells also formed large granules in the cytoplasm (Fig. 1b, white arrow). This result suggests that overexpression of RBM45 leads to cytoplasm accumulation in granules, similar to the RBM45 inclusions observed in ALS patient cells.

To identify the sequence(s) responsible for nuclear localization, we analyzed the amino acid sequence of RBM45 using the computational program cNLS Mapper^23^ and identified two potential nuclear localization sequences (NLSs), 376-PSCKKKAPAE-385 and 454-RLKVMLADSPREESNKRQR-472, in the C-terminal region (Fig. 1c). Within the second predicted NLS (454–472), the sequence 469-KRQR-472 matches the consensus NLS sequence K-K/R-X-K/R, which is rich in basic amino acids^24^. We then examined these two putative NLSs for RBM45 nuclear localization by mutagenesis and subcellular fractionation. Recombinant HA-tagged RBM45 mutants, M1 (379-AAA-381) and M2 (469-AAQA-472), that had the basic amino acids in the first and second predicted NLSs substituted with alanine were expressed in the Neuro2A cells (Fig. 1c). Subcellular fractionation and immunoblot showed that only 38.9% of HA-RBM45 M2 mutant protein was located in the nucleus, compared to the 82.5% of the wild-type protein and 76.0% of the M1 mutant protein (Fig. 1d). A double mutant M1/2 that included both M1 and M2 mutations exhibited 39.0% nuclear localization, similar to the M2 mutant alone (Fig. 1d). Therefore, the sequence 379-KKK-381 in the first NLS candidate did not appear to function in RBM45 nuclear localization. Immunofluorescence staining confirmed that the HA-RBM45 M2 mutant had increased cytoplasmic retention compared to wild-type HA-RBM45 (Fig. 1e). These results indicate that the sequence 469-KRQR-472 in the second candidate NLS is the primary determinant of the nuclear localization of RBM45.

Within the second NLS candidate (454–472) are additional basic residues (454R, 456K and 464R) that may contribute to RBM45’s nuclear localization (Fig. 1c). To examine these residues for a role in RBM45 nuclear localization, we generated the HA-RBM45 M3 mutant in which 454R, 456K and 464R were each substituted with alanine (Fig. 1f). A double mutant M2/3 that contained both M2 and M3 mutations was also included in the subcellular fractionation assay. Surprisingly, the HA-RBM45 M3 mutant and M2/3 double mutant showed 45.8% and 11.7% nuclear localization, respectively (Fig. 1g). We thus conclude that both clusters of basic amino acids in the NLS (454–472) contribute to nuclear localization of RBM45. Moreover, five of the six basic amino acids, 454R, 456K, 469K, 470R and 472R, in the RBM45 NLS are highly conserved across metazoa, supporting their crucial function in the nuclear localization of RBM45 (Supplementary Fig. S1d). Taken together, we conclude that RBM45 has a bipartite NLS containing two clusters of basic amino acids necessary for nuclear localization.

### Identification of RBM45 domains required for self-association

**Figure 2:**
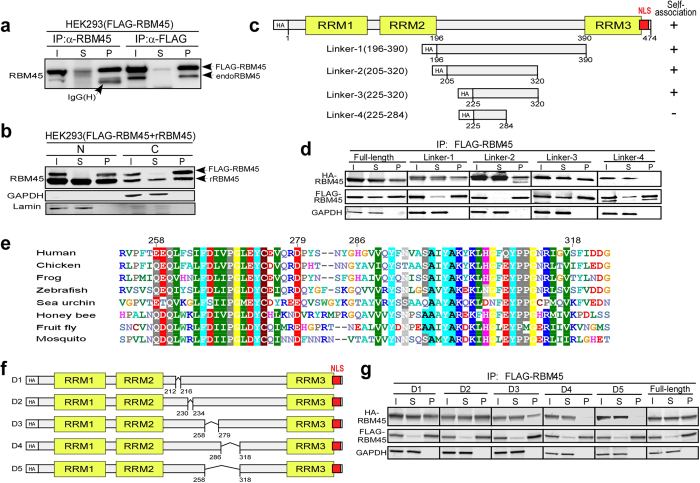
Self-association of RBM45. (**a**) Co-immunoprecipitation of endogenous RBM45 with FLAG-RBM45 in HEK293 cells. FLAG-RBM45 was expressed in HEK293 cells and immunoprecipitated with RBM45 C-terminal antibody (epitope residues 460–474) or FLAG antibody. The IP fractions were immunoblotted with RBM45 antibody against an internal region (epitope residues 216–265). The bands of the FLAG-RBM45 (56.6 kD) and endogenous RBM45 (53.3 kD) are indicated by arrows. The IgG-heavy chain that cross-reacts with the secondary antibody used in this experiment is also denoted. I = Input, S = Supernatant, P = Pellet. (**b**) RBM45 self-association occurs in both the nucleus and cytoplasm. FLAG-RBM45 and recombinant untagged-RBM45 (rRBM45) were co-expressed into HEK293 cells. Cells were fractionated followed by immunoprecipitation with FLAG antibody. The IP fractions were immunoblotted with RBM45 C-terminal antibody, GAPDH (cytoplasmic marker) and Lamin A/C (nuclear marker) antibodies. The bands of the FLAG-RBM45 (56.6 kD) and rRBM45 (53.3 kD) are indicated by arrows. N = Nuclear extract fraction, C = Cytoplasmic extract fraction. (**c**) Schematic of HA-RBM45 linker truncation constructs. The constructs capable of self-association are indicated by “+” on the right. (**d**) Co-immunoprecipitation of HA-RBM45 linker truncation constructs with full-length FLAG-RBM45 in HEK293 cells. HA-RBM45 constructs from **c** were transfected into HEK293 cells that stably express the full-length FLAG-RBM45. FLAG-IP were performed and the IP fractions were immunoblotted with HA (HA-RBM45 constructs), FLAG (FLAG-RBM45) and GAPDH (negative IP control) antibodies. (**e**) Protein alignment of the RBM45 revealing the two clusters of amino acid residues (258–279 and 286–318) that are highly conserved within the linker region between RRM2 and RRM3. Conserved residues (including homologous residues) are highlighted (conservation threshold = 80%). (**f**) Schematic of HA-RBM45 deletion constructs that were tested in **g**. The angled lines show the amino acid residues that are internally deleted. (**g**) Co-immunoprecipitation of HA-RBM45 deletion constructs with full-length FLAG-RBM45 in HEK293 cells demonstrating that the region 258–318 is important for self-association. Transfection and immunoprecipitation were performed as described in **d**.

ALS-linked proteins TDP-43 and FUS contain prion-like domains and are intrinsically prone to aggregation^25^. Furthermore, dimerization of TDP-43 is believed to nucleate protein aggregation leading to inclusions *in vivo*^26^,^27^,^28^. Similar to TDP-43 and FUS, RBM45 forms intracellular inclusions in ALS and FTLD patients^20^. Therefore we sought to determine whether RBM45 self-associates to form a dimer or multimer. Recombinant FLAG-tagged RBM45 was expressed in HEK293 cells and examined for interactions with endogenous RBM45 proteins by co-immunoprecipitation (co-IP). The co-IP analysis was performed using whole cell lysate and antibodies against the FLAG or the RBM45 C-terminal epitope, followed by immunoblot using an antibody against an internal epitope (residues 216–265) of RBM45 (See Methods). The results indicated that FLAG-tagged RBM45 efficiently co-purified with endogenous RBM45 (Fig. 2a). Since RBM45 is predominately localized in the nucleus (Fig. 1a), we asked if the RBM45 self-association is specific to the nuclear protein. We performed co-IP assay using either the nuclear or cytoplasmic fraction of HEK293 cells that expressed both untagged and the FLAG-tagged recombinant RBM45 proteins. Anti-FLAG IP showed that the untagged recombinant RBM45 co-purified with FLAG-RBM45 from both the nuclear and cytoplasmic fractions (Fig. 2b).

We next sought to map the domain responsible for RBM45 self-association by screening a series of truncated HA-tagged RBM45 fragments for interaction with the full-length FLAG-RBM45 protein co-expressed. We initially focused on the uncharacterized linker region between RRM2 and RRM3 by expressing four HA-RBM45 truncation constructs, Linker-1, -2, -3 and -4 (Fig. 2c), in a HEK293 stable cell line. Anti-FLAG IP assay was performed to identify the HA-RBM45 fragments that co-purified with FLAG-RBM45. Among all constructs examined, only Linker-4 failed to co-purify with FLAG-RBM45, suggesting that the region between residues 284 and 320 is necessary for self-association (Fig. 2d). Within this linker region between RRM2 and RRM3, we identified two motifs (258–279 and 286–318) with sequences highly conserved across species (Fig. 2e). To test these two motifs for self-association, we generated HA-RBM45 constructs, D1 to D5, with specific deletions in the linker region (Fig. 2f). HA-RBM45 constructs D1 (Δ212–216) and D2 (Δ230–234) with non-conserved residues deleted serve as negative controls. Constructs D3 (Δ258–279) and D4 (Δ286–318) had individual motifs removed, while D5 (Δ258–318) had both motifs removed (Fig. 2f). Anti-FLAG co-IP demonstrated that HA-RBM45 mutant D3 exhibited reduced co-IP, while HA-RBM45 mutants D4 and D5 failed to co-purify with FLAG-RBM45 when compared to D1, D2 or full-length RBM45 (Fig. 2g). Therefore, we concluded that the two conserved motifs, 258–279 and 286–318, within the linker region are crucial for RBM45 self-association, whereas all three RRMs are not required for self-association.

### RBM45 forms oligomers

**Figure 3:**
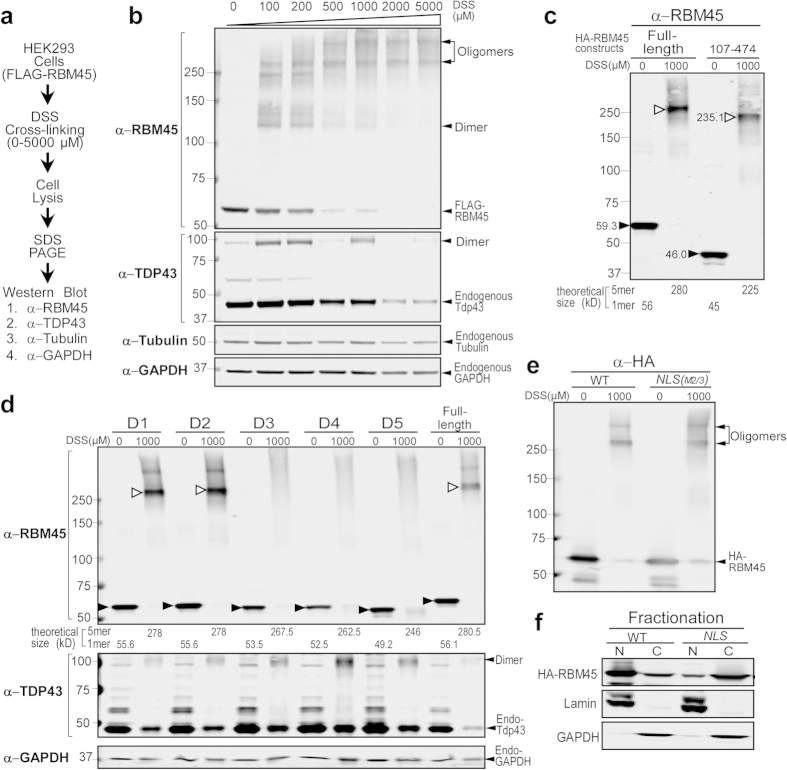
RBM45 forms high-molecular-weight oligomers. (**a**) Diagram of the DSS crosslinking experiments. Live HEK293 cells constitutively expressing FLAG-RBM45 underwent *in vivo* crosslinking using the cell-permeable NHS-ester crosslinker DSS with increasing concentrations from 0 to 5000 μM. The cells were lysed and lysates were run on denaturing SDS-PAGE and probed for immunoblot. (**b**) Immunoblot analysis of samples generated from **a**. Samples probed with RBM45 C-terminal antibody showed that FLAG-RBM45 in non-crosslinked cells migrates at the expected molecular weight of a monomer (56 kD), whereas FLAG-RBM45 in DSS-treated cells migrates at the molecular weight of a dimer (~110 kD) or a pentamer (~280 kD). The arrows indicate the sizes of FLAG-RBM45 monomer and oligomers. Samples probed with TDP-43 antibody revealed the TDP-43 monomer (45 kD) and TDP-43 dimer (~90 kD). Samples probed with β-tubulin and GAPDH antibodies were negative control to rule out non-specific crosslinking of cellular monomeric proteins. (**c**) HA-tagged full-length RBM45 and RRM1-deletion construct (107–474) were expressed in HEK293 cells and DSS *in vivo* crosslinking (1 mM DSS for 10 minutes) were performed. The oligomerization of the HA-RBM45 constructs was examined by immunoblot and probed with RBM45 C-terminal antibody. Filled arrowheads indicate the monomers, while open arrowheads indicate the pentamers. The observed molecular weights of the monomers and pentamers are indicated next to the bands. The theoretical molecular weights of the monomers and pentamers are indicated below the blot. (**d**) The HA-tagged D1-D5 constructs (Fig. 2f) were tested for their ability to oligomerize as described in **c**. Filled arrowheads indicate the monomers, while open arrowheads indicate the pentamers. The theoretical molecular weights of the monomers and pentamers are indicated below the blot. The blot was also probed with TDP-43 (crosslinking positive control) and GAPDH (negative control) antibodies. Full length gels are shown in Supplemental Figure S2b. (**e**) RBM45 homo-oligomerization occurs in both the nucleus and cytoplasm. The oligomerization of HA-tagged RBM45 or the NLS M2/3 mutant (from Fig. 1) was examined by immunoblot and probed with HA antibody. Arrowheads denote HA-RBM45 and oligomer species. (**f**) Subcellular fractionation of the HA-RBM45 constructs in **e**. Fractionation was performed as described in Fig. 1d.

To determine if RBM45 self-association leads to dimerization or oligomerization, we performed *in vivo* cross-linking with disuccinimidyl suberate (DSS) to capture native RBM45 assemblies in live cells. DSS is an irreversible, cell membrane permeable cross-linking reagent containing bifunctional amine-reactive NHS-esters^29^. We first treated the HEK293 cells that stably express FLAG-RBM45 with a titration of DSS from 0 to 5000 μM and analyzed the FLAG-RBM45 assembly states by SDS-PAGE and immunoblot (Fig. 3a). On SDS-PAGE, FLAG-RBM45 from untreated cells migrated as monomer with the expected molecular weight of 56 kD, whereas the cross-linked FLAG-RBM45 from the cells treated with up to 5 mM DSS migrated as larger species with expected molecular weights for both the dimer and higher ordered species with molecular weight above 250 kD (Fig. 3b). Of the higher mass oligomer species, the size of the smaller band was estimated to be ~266 kD by resolving the samples on a high-resolution Tris-Acetate 3–8% gradient SDS-PAGE gel along with high-molecular-weight size markers (Supplementary Fig. S2a). A previous report suggested that TDP-43 forms a homo-dimer upon cross-linking^28^. Similarly, we also observed homo-dimer formation of TDP-43, but not β-tubulin or GAPDH, upon treatment with 5 mM DSS (Fig. 3b and Supplementary Fig. S2b), suggesting our experimental conditions effectively captured protein assembly states. If RBM45 forms homo-oligomers, the 280 kD (theoretical size) product likely represents a pentamer of FLAG-RBM45. The 280 kD cross-linked product appeared to be stable and did not continue to form larger species when treated with 5 mM DSS for an extended time up to 50 minutes (Supplementary Fig. S2c). In addition to analyzing the oligomerization of overexpressed recombinant FLAG-RMB45, we have also observed similar oligomer formation of endogenous RBM45 in HEK293 and SK-N-SH cells upon DSS treatment (Supplementary Fig. S2d).

To determine the stoichiometry of RBM45 homo-oligomers, we analyzed the size of the cross-linked product of a truncated HA-RBM45 variant (107–474) that lacks RRM1. By SDS-PAGE, the monomer of the full-length and truncated HA-RBM45 migrated at 59 and 46 kD, similar to the calculated 56 and 45 kD, respectively (Fig. 3c, filled arrowhead). Upon DSS treatment, the cross-linked products of the truncated HA-RBM45 (107–474) migrated at 235 kD, similar to the calculated 225 kD of a homo-pentamer (Fig. 3c, open arrowhead). Therefore, the mass of the cross-linked products of the truncated HA-RBM45 suggested that RBM45 forms a homo-pentamer, though further studies are necessary to determine if other proteins are also incorporated into this oligomeric species.

Next, we examined if RBM45 oligomerization is mediated by amino acids 258–279 and 286–318 that are required for RBM45 self-association. RBM45 deletion mutants, D1-D5, were expressed in HEK293 cells and treated with DSS. Indeed, mutants D3, D4 and D5 that failed to self-associate also failed to form high-molecular-weight oligomers (Fig. 3d). Mutants D1 and D2 formed homo-pentamer upon cross-linking, similar to the full-length RBM45 (Fig. 3d). Thus, the region spanning from residue 258 to 318 is responsible for RBM45 homo-oligomerization and termed the homo-oligomer assembly (HOA) domain.

Lastly, we determined in which cell compartment the RBM45 oligomerization occurs. We compared the oligomerization propensity of wild-type RBM45 that predominately localized in the nucleus with the RBM45 NLS-M2/3 mutant that was predominately retained in the cytoplasm (Fig. 1g). Interestingly, both the wild-type RBM45 and the NLS-M2/3 mutant demonstrated comparable levels of homo-pentamers upon cross-linking (Fig. 3e,f), suggesting that differential subcellular localization of RBM45 does not affect oligomerization. Moreover, subcellular fractionation of the cells following DSS cross-linking showed that RBM45 is present as homo-oligomers in both the cytoplasm and the nucleus (Supplementary Fig. S2e). By contrast, TDP-43 dimerization occurs predominately in the nucleus in these experiments.

### RBM45 associates with ALS-linked proteins

**Figure 4:**
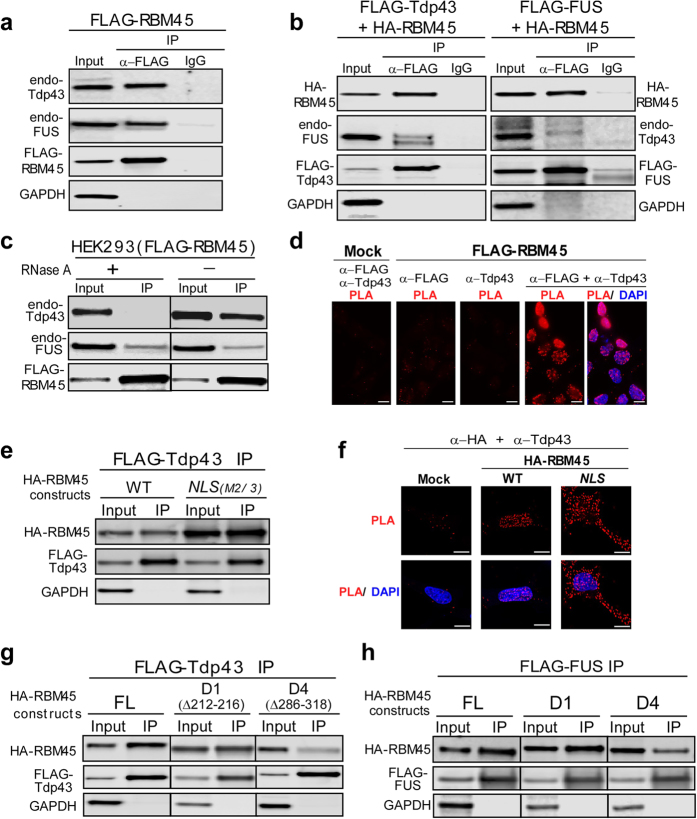
RBM45 interacts with TDP-43 and FUS. (**a**) FLAG-RBM45 was expressed in HEK293 cells and immunoprecipitations with FLAG antibody or IgG were performed. Live cells were treated with 0.1% formaldehyde to cross-link proteins prior to cell lysis and immunoprecipitation. The crosslinking was reversed by heating in SDS-sample buffer prior to SDS-PAGE. The IP fractions were immunoblotted with TDP-43, FUS, FLAG (FLAG-RBM45) and GAPDH (negative control) antibodies. (**b**) HA-RBM45 was transfected into HEK293 cells stably expressing FLAG-TDP-43 (left) or FLAG-FUS (right). Immunoprecipitations and immunoblot were performed as described in **a**. HA-RBM45 was detected with HA antibody. (**c**) In-cell RNase treatment and crosslinking-IP were performed on cells expressing FLAG-RBM45. The amount of TDP-43, but not FUS, that co-purified with FLAG-RBM45 was reduced with the RNase treatment. (**d**) In-cell FLAG-RBM45 and TDP-43 interactions as demonstrated by Proximity Ligation Assay (PLA) in the nucleus of HEK293 cells. PLA was performed on HEK293 cells stably expressing FLAG-RBM45. Each red dot indicates the protein-protein interaction (<40 nm) between FLAG-RBM45 and endogenous TDP-43. The primary antibodies are listed on top of each panel. DAPI was used as the nuclear stain. Scale bar: 10 μM. (**e**) Wild-type and cytoplasmic retained RBM45 have similar binding efficiency with TDP-43. HA-tagged RBM45 wild-type or the NLS M2/3 mutant was transfected into the HEK293 cell line stably expressing FLAG-TDP-43. FLAG-TDP-43 immunoprecipitation and immunoblot were performed as described in **b**. (**f**) PLA assays showing the in-cell protein-protein interactions between HA-RBM45 wild-type or NLS M2/3 mutant and endogenous TDP-43 in HEK293 cells. HA-RBM45 constructs were expressed in HEK293 cells and PLA assays were performed. DAPI was used as the nuclear stain. Scale bar: 10 μM. (**g**) The self-association deficient RBM45 exhibits reduced binding to TDP-43. HA-tagged full-length, D1 and D4 RBM45 constructs were transfected into the HEK293 cell line stably expressing FLAG-TDP-43. FLAG-TDP-43 immunoprecipitation and immunoblot were processed as in **b**. (**h**) The self-association deficient RBM45 exhibits reduced binding to FUS. HA-tagged full-length, D1 and D4 RBM45 constructs were transfected into the HEK293 cell line stably expressing FLAG-FUS. FLAG-FUS immunoprecipitation and immunoblot were processed as described in **b**.

As noted above, RBM45 and TDP-43 co-localize to cytoplasmic inclusions in ALS and FTLD patients. To determine whether incorporation of RBM45 and TDP-43 into inclusion bodies is mediated by direct interaction between RBM45 and TDP-43 or other ALS-linked proteins, we expressed FLAG-RBM45 protein in HEK293 cells and performed co-IP. To detect transient or weak interactions, the cells were treated with formaldehyde to cross-link associated proteins prior to co-IP analysis. Anti-FLAG co-IP assay demonstrated that both endogenous TDP-43 and FUS co-purified with FLAG-RBM45 (Fig. 4a). Reciprocal co-IP analysis using cells co-expressing FLAG-TDP-43 and HA-RBM45 showed that FLAG-TDP-43 co-purified with HA-RBM45 as well as endogenous FUS protein (Fig. 4b, left panel). Similarly, IP of FLAG-FUS co-purified with HA-RBM45 and endogenous TDP-43 protein (Fig. 4b, right panel).

TDP-43 and FUS are both RNA-binding proteins containing RRM domains^20^,^30^,^31^. Since RBM45 contains three RRM domains, it may associate with TDP-43 and FUS through RNA-protein interactions. To determine if the associations between RBM45 and ALS-linked proteins are RNA-dependent, we developed an in-cell RNase treatment assay with which the cellular RNA is degraded by RNase prior to the formaldehyde cross-linking and anti-FLAG IP. Interestingly, the in-cell RNase treatment significantly reduced the amounts of endogenous TDP-43 co-purified with FLAG-RBM45, while the FUS-RBM45 association was resistant to in-cell RNase treatment (Fig. 4c). This result suggests that the interaction between RBM45 and TDP-43 is RNA-dependent, while the FUS-RBM45 association is mediated by direct protein-protein interactions.

RBM45, TDP-43 and FUS are localized predominately in the nucleus in healthy cells and form cytoplasmic inclusions in ALS/FTLD patient cells. To determine if endogenous proteins located in the nucleus interact, we performed co-IP analysis of endogenous proteins in human neuroblastoma SK-N-SH cells. Endogenous TDP-43, but not endogenous FUS, co-purified with endogenous RBM45 (Supplementary Fig. S3a), indicating that TDP-43 interacts with RBM45 at endogenous protein levels.

To determine whether the associations between RBM45 and ALS-linked proteins occur in the nucleus, cytoplasm or both, we employed the proximity ligation assay (PLA) to visualize the co-localization of RBM45 and its binding proteins. PLA visualizes protein-protein interactions *in situ* by detecting two proteins of interest with specific primary antibodies raised in different species followed by binding of two species-specific DNA-linked secondary antibodies, called the PLA probes. When the two PLA probes are within 40 nm of close proximity, ligation and polymerase amplification of the two DNA strands allows highly sensitive detection of the co-localized proteins^32^. Using HEK293 cells expressing FLAG-RBM45, the PLA assay generated strong signals when both anti-FLAG and anti-TDP-43 antibodies were used (Fig. 4d). When only a single primary antibody or vector-transfected cells was used, the PLA signals were at background level. Thus, we conclude that RBM45 and TDP-43 are closely associated in the nucleus.

To further determine if RBM45 and TDP-43 associate in the cytoplasm, we co-expressed FLAG-TDP-43 together with the HA-RBM45-NLS mutant that predominately accumulates in the cytoplasm, followed by co-IP to detect physical interactions between RBM45 and TDP-43. We observed significant association between the RBM45-NLS mutant and TDP-43 (Fig. 4e). Similarly, the PLA assay detected a strong association between the HA-RBM45-NLS mutant and endogenous TDP-43 in the cytoplasm (Fig. 4f). Therefore, we conclude that RBM45 associates with TDP-43 when retained in the cytoplasm. This raised the question whether the RBM45-NLS mutant induced the cytoplasmic retention of TDP-43. Subcellular fractionation, however, showed that the overexpression of the RBM45-NLS mutant did not significantly alter the nuclear localization of endogenous TDP-43 (Supplementary Fig. S3b), indicating that RBM45 does not participate in the shuttling of TDP-43 between the nucleus and cytoplasm.

### RBM45 oligomerization mediates association with ALS-linked proteins

We next asked whether the oligomerization of RBM45 is also necessary for its association with TDP-43 and FUS. Anti-FLAG co-IP analysis using cells co-expressing FLAG-TDP-43 and HA-RBM45 deletion mutants, D1 and D4, showed that wild-type and the D1 mutant HA-RBM45 co-purified with FLAG-TDP-43 (Fig. 4g). However, the efficiency of RBM45-TDP-43 co-IP was greatly reduced for the HA-RBM45 D4 mutant, which fails to form homo-oligomer. Similarly, FLAG-tagged FUS also failed to effectively co-IP with the HA-RBM45 D4 mutant (Fig. 4h). Reciprocal co-IP assay using either the full-length FLAG-RBM45 or the D4 mutant showed that the D4 mutant exhibited significantly reduced binding to endogenous TDP-43 and FUS (Supplementary Fig. S3c). These results suggest that RBM45 oligomerization is important for mediating interactions with the ALS-linked proteins TDP-43 and FUS.

Since the RBM45-TDP-43 association is RNA-mediated, we examined the importance of the three RRM domains of RBM45 for TDP-43 association. We performed co-IP assays of FLAG-TDP-43 with different RBM45 constructs that lack individual RRM domains (Supplementary Fig. S3d). Interestingly, removal of individual RRM domains in RBM45 mutants ΔRRM1, ΔRRM1/2 and ΔRRM3 only marginally reduced association with FLAG-TDP-43 (Supplementary Fig. S3e). In contrast, the mutant ΔHOA-RRM3, which had the HOA domain as well as RRM3 removed, failed to co-IP with FLAG-TDP-43 (Supplementary Fig. S3e). Taken together, these data suggests that a combination of the HOA domain and at least RRM3 mediates RBM45 association with TDP-43.

### Cytoplasmic RBM45 mutant is recruited to stress granules

**Figure 5:**
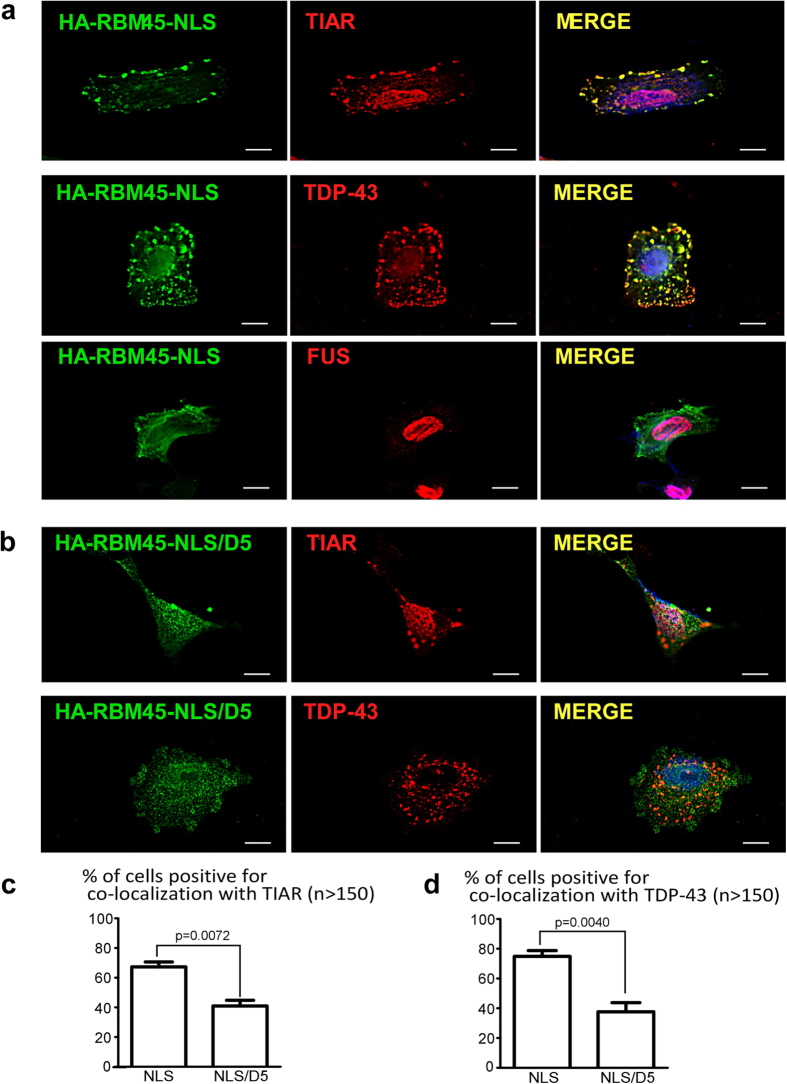
RBM45 homo-oligomerization mediates its incorporation into stress granules and TDP-43 association. (**a**) The cytoplasmic granules containing the RBM45 HA-NLS mutant are immunoreactive with stress granule markers TIAR (top) as well as TDP-43 (middle), but are not immunoreactive with FUS (bottom). HA-RBM45 NLS M2 mutant (from Fig. 1) was transfected into SK-N-SH cells, and 48 hr post-transfection, the transfected cells were stressed with 1 mM sodium arsenite for 30 minutes followed by immunostaining. HA-RBM45 NLS was immunostained with rabbit anti-HA antibody (green). Nuclei were labeled with DAPI. Scale bar: 10 μm. TIAR was stained with mouse monoclonal anti-TIAR antibody, TDP-43 was stained with mouse monoclonal anti-TDP-43 antibody that recognizes full-length as well as the cleaved C-terminal fragment of TDP-43, and FUS was stained with mouse monoclonal anti-FUS antibody. (**b**) RBM45-NLS mutant with additional HOA domain deletion (NLS/D5 construct) are not incorporated into cytoplasmic granules and fail to co-localize with TIAR (top) or TDP-43 (bottom). Transfection and immunofluorescence experiments were performed as described in **a**. (**c**,**d**) Quantification demonstrating the percentage of the cells transfected with NLS or NLS/D5 constructs that form cytoplasmic granules positive for TIAR in **c** or TDP-43 in **d** (n = 3; cell numbers > 50 per experiment). The *p*-value was calculated by two-tailed paired t-test. Error bar represents SEM.

Oxidative stress has been implicated in the pathogenesis of ALS and FTLD^2^,^33^,^34^. Upon oxidative insult, both TDP-43 and FUS translocate from the nucleus and incorporate into cytoplasmic stress granules^9^,^10^,^11^. We recently demonstrated that RBM45 is similarly translocated into cytoplasmic stress granules when cells are exposed to oxidative stress^35^. Given the physical association of RBM45 and ALS-linked RNA-binding proteins (Fig. 4), we asked whether cytoplasmic RBM45 is recruited to stress granules similar to TDP-43 and FUS. We expressed the HA-RBM45 NLS mutant M2 that exhibits predominately cytoplasmic retention in human SK-N-SH neuroblastoma cells and stressed the cells with 1 mM sodium arsenite for 30 minutes. Immunofluorescence microscopy for HA-RBM45 showed that approximately 70% of the cells transfected with the RBM45-NLS mutant formed distinct large cytoplasmic granules (Fig. 5a), while only about 15% of the cells transfected with wild-type RBM45 formed cytoplasmic granules. Co-localization using an antibody specific to the stress granule marker protein (TIA-1 related protein) demonstrated that cytoplasmic RBM45 was localized to stress granules upon sodium arsenite treatment (Fig. 5a, top panel). RBM45 cytoplasmic stress granules contained TDP-43 (Fig. 5a, middle panel), but not FUS (Fig. 5a, bottom panel and Supplementary Fig. S4). This result recapitulates our prior *in vivo* findings that RBM45 was found in the TDP-43-containing but not FUS-containing inclusions, in neurons of ALS and FTLD patients^20^. When the HOA domain was deleted in the NLS/D5 mutant (Fig. 5b), significantly fewer cells contained cytoplasmic granules positive for TIAR (Fig. 5b, top panel and Fig. 5c) or TDP-43 (Fig. 5b, bottom panel and Fig. 5d). These data suggest that the oligomerization of RBM45 in the cytoplasm mediates its incorporation into stress granules as well as its association with TDP-43 in stress granules.

## Discussion

In both ALS and FTLD, aggregation of RNA-binding proteins contributes to disease pathogenesis and generates neuropathologic inclusions within the brain and spinal cord. RBM45 was first described as a neuronal RNA-binding protein expressed under spatiotemporal control in the rat brain^21^. We previously observed elevated levels of RBM45 in the CSF of ALS and FTLD patients and intracellular inclusions of RBM45 in neurons and glia of ALS and FTLD patients^20^. In this report, we study the domain structure and protein-protein interactions of RBM45, providing mechanistic insights into the cellular functions of RBM45 relevant to ALS and FTLD.

**Figure 6:**
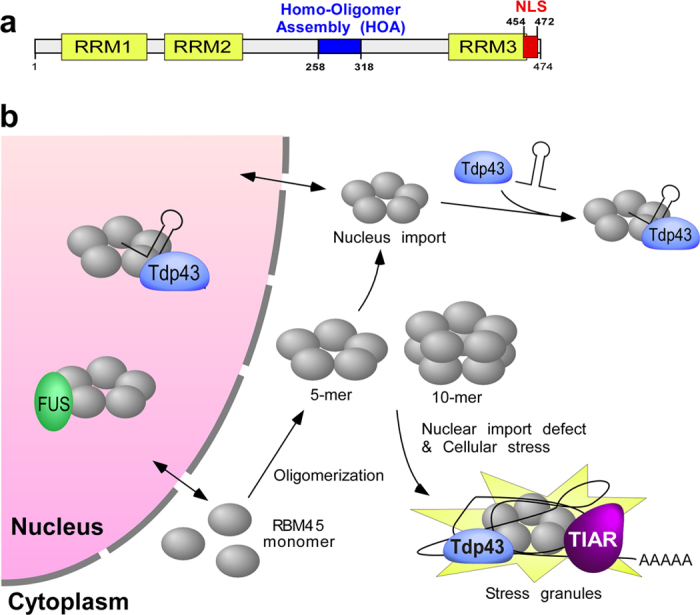
Schematic overview of RBM45 domains structure and protein-protein interactions. (**a**) Domain structure of RBM45 with the newly identified HOA (homo-oligomer assembly) domain highlighted in blue and the NLS in red. (**b**) A working model for RBM45 homo-oligomerization and distribution in the cell. RBM45 monomers assemble into a ring-shape pentamer which may dimerize to form a decamer. The RBM45 homo-oligomer acts as a scaffold to associate with TDP-43 mediated by RNA in the nucleus or cytoplasm. Accumulation of RBM45 in the cytoplasm leads to stress granule inclusion together with TIAR and TDP-43.

We identified a novel HOA domain (residues 258–318) within RBM45 that is responsible for self-association and oligomerization (Fig. 6a). The HOA domain is well-conserved across species, suggesting an important functional role. Indeed, the HOA domain confers both structural (i.e. oligomerization) and functional (i.e. association with ALS-linked proteins) attributes of RBM45. Moreover, we estimate the stoichiometry of the RBM oligomer to be a homo-pentamer and decamer (Fig. 3e), though further studies are required to confirm this proposed homo-oligomer structure. We propose that RBM45 forms a closed ring-like pentamer (Fig. 6b), as this pentamer is stable without an increase of molecular weight when treated with increasing amounts of DSS or extended incubation time. Since the RBM45 homo-oligomers were observed in both the cytoplasm and nucleus, the pentamer may be assembled in the cytoplasm and imported into the nucleus. Alternatively, RBM45 may be imported into the nucleus as a monomer prior to oligomerization.

Many known RNA chaperones contain RRM domains^36^,^37^,^38^ and/or function in the form of oligomers^39^,^40^. We suspect that RBM45 function as an RNA chaperone, as its interaction with TDP-43 is mediated by RNA and requires RBM45 homo-oligomerization. TDP-43 and FUS both exert their biological functions in the nucleus, regulating multiple RNA metabolic pathways. Interactions between oligomeric RBM45 with TDP-43 or FUS may modulate their association with distinct nuclear sub-structures that confer specific RNA metabolic processes. Although cytoplasmic RBM45 associates with TDP-43, RBM45 does not modulate the shuttling of TDP-43 between the nucleus and cytoplasm. The cytoplasmic interactions between RBM45 and TDP-43 may occur prior to or concurrent with the formation of stress granules in response to cell stress. Recently, Hans *et al.* reported that RBM45 binds to overexpressed C-terminal fragment (CTF) of TDP-43 in mammalian cells^22^, which is consistent with our results that RBM45 binds to endogenous full-length TDP-43. We suspect that TDP-43 interacts with RBM45 via the glycine-rich domain located within its CTF, as this glycine-rich domain has been shown to mediate the interactions between TDP-43 and other hnRNP proteins^41^. Determining whether RBM45 homo-oligomers facilitate the ribonucleoprotein complexes assembly of other ALS-linked proteins may provide new mechanistic insights into normal and pathological RNA metabolism.

Both TDP-43 and FUS bind pre-mRNA with long introns^42^,^43^,^44^,^45^, and a subset of RNA targets are bound and regulated jointly by both proteins^42^. Three of these regulated genes (*PARK2, SMYD3 and KCNIP4*) are important for neuronal function and are down-regulated in sporadic ALS motor neurons harboring TDP-43 pathology, suggesting a shared mechanism of neurodegeneration caused by dysregulation of TDP-43 or FUS. As RBM45 and TDP-43 interactions are RNA-dependent, it suggests that RBM45 and TDP-43 share the regulation of specific RNA targets. Future studies are needed to identify the RNA targets bound by RBM45 to determine RNA targets co-regulated by RBM45 and TDP-43.

Although RBM45 is a nuclear protein, overexpressed wild-type RBM45 can form cytoplasmic granules that resemble the inclusions observed in ALS/FTLD patient tissues. Disruption of the RBM45 NLS and cellular stress further exacerbate cytoplasmic accumulation and lead to increased granule formation that co-localize with TDP-43 and stress granule marker TIAR (Fig. 6b). In cells that express the homo-oligomerization deficient RBM45, significantly fewer RBM45-containing stress granules are observed, indicating that RBM45 oligomerization mediates its association with TDP-43 and stress granules. Stress granules consist of protein-RNA complexes in which mRNA is translationally inactive to prioritize protein synthesis in response to the cellular stressor^2^. Therefore, the co-localization of RBM45 and TDP-43 in stress granules is consistent with the RNA-dependent interaction between these two proteins. Several RNA-binding proteins commonly found in stress granules, including TDP-43, have been genetically linked to neurodegeneration^33^. While genetic alterations of RBM45 have not yet been identified in neurologic diseases, the physical association of RBM45 with ALS-linked proteins suggests that it may play a role in regulating the normal function of these proteins in RNA metabolism. We did not detect RBM45 in FUS-containing inclusions, consistent with observations in ALS and FTLD patient tissues^20^. Similarly, TDP-43 and FUS do not co-localize in cytoplasmic inclusions^7^,^13^,^34^. We propose that RBM45 and TDP-43 converge on the same pathogenic mechanism that is not shared with FUS. Interestingly, the NLSs in RBM45 and TDP-43 are both classical NLS sequences, which are recognized by Importin α/β and adopt the Imp α/β pathway for nuclear transport. Nuclear import defects of TDP-43 have been reported in aging brains, resulting in cytoplasmic mislocalization of TDP-43^14^. Disruption of this shared nuclear import pathway during aging or disease may result in the cytoplasmic retention of both proteins, leading to their interaction and accumulation in stress granules. In addition, a naturally occurring alternative isoform of RBM45 is predicted to contain the HOA domain but lacks the final 147 amino acids that contain the RRM3 and NLS sequences. This RBM45 isoform is predicted to localize to the cytoplasm and could localize to stress granules under stress conditions. While we have not detected this alternative isoform in our *in vitro* studies, future studies will determine the presence or absence of this alternative protein isoform in ALS and other neurologic diseases.

We have identified structural domains in RBM45 that contribute to its function, subcellular localization, homo-oligomerization and its interactions with the ALS-associated proteins TDP-43 and FUS. Homo-oligomerization of RBM45 is mediated by a novel HOA domain that is conserved across species and important for its protein-protein interactions and association with cytoplasmic stress granules. These results provide new insights into the role that protein assemblies play in the normal and pathological functions of RNA-binding proteins and provide further evidence of the association of RBM45 and TDP-43 in both the nucleus and cytoplasm, a phenomenon first observed in cytoplasmic inclusions in ALS/FTLD patients.

## Materials and Methods

### Plasmid construction and mutagenesis

The plasmids of RBM45, TDP-43 and FUS cDNA clones, cGST-hRBM45 (HsCD00356971), pDONR221-TDP-43 (HsCD00079870), and pDNR-Dual-FUS (HsCD00002882), were obtained from the DNASU Plasmid Repository at Arizona State University, Tempe. The cDNA was amplified by PCR using Phusion High-Fidelity DNA Polymerase (NEB) and sub-cloned into the pcDNA3 vector (Invitrogen). The 3xFLAG tag (DYKDHDGDYKDHDIDYKDDDDK) or 2xHA tag (DYPYDVPDYAGGAAYPYDVPDYA) was appended to the N-terminus of specific proteins to generate the 3xFLAG- or 2xHA-tagged construct. The EGFP-RBM45 construct was generated by inserting the EGFP gene into the N-terminus of RBM45 gene in pcDNA3. The mutations were introduced by site-directed mutagenesis using overlap extension PCR^46^. The sequences of the constructs were confirmed by DNA sequencing and the sizes of the expressed proteins confirmed by immunoblot.

### Cell culture

HEK293 (FreeStyle™ 293-F Cells, Invitrogen), Neuro2A (ATCC) and SK-N-SH (Sigma) cells were cultured in DMEM medium with 10% FBS and 1% Pen-Strep at 37 °C with 5% CO_2_. SHSY5Y cells were cultured in DMEM/F12 medium with 15% FBS, 1% non-essential amino acids and 1% Pen-Strep at 37 °C with 5% CO_2_. Transfection was performed using the Lipofectamine 2000 (Life technologies) and transfected cells were harvested 48 or 72 hours post-transfection. Stable cell lines were selected in the presence of 500 μg/ml G418 (Life Technologies).

### Subcellular fractionation

Cells were grown on 6-well plates till confluent and harvested. The cell pellet was gently resuspended in 150 μl cytoplasmic extraction buffer (10 mM HEPES pH7.6, 60 mM KCl, 1 mM EDTA, 0.15% NP40, 0.5 mM DTT and protease/phosphatase inhibitors) and incubated on ice for 5 min. The cells were spun at 600 × g for 4 min at 4 °C and the supernatant was transferred to a new tube as crude cytoplasmic fraction (~150 μl). The pellet was gently washed with 100 μl cytoplasmic extraction buffer without NP40 and spun at 600 × g at 4 °C for 4 min. The supernatant was discarded and the pellet was resuspended with 150 μl nuclear extraction buffer (20 mM HEPES pH 7.9, 1.5 mM MgCl2, 430 mM NaCl, 0.2 mM EDTA, 25% glycerol and protease inhibitors) and incubated at 4 °C with rotation for 15 min. The nuclear extract and the crude cytoplasmic fraction were cleared by centrifugation at 16,000 × g at 4 °C for 10 min. Equal volume of the nuclear and cytoplasmic fractions were analyzed by SDS-PAGE gel and immunoblot. The band intensity was quantified with Image Studio software (LiCOR) and the relative abundance of the nuclear RBM45 in total RBM45 was calculated as [(nuclear fraction)/(nuclear fraction + cytoplasmic fraction)] × 100%. Statistical analysis was performed using two-tailed paired t-test.

### Immunofluorescence

Cells were grown on Poly-D-Lysine coated coverslips to 60% confluent. Fixation was performed with 4% paraformaldehyde at room temperature for 15 min, followed by permeabilization with 0.2% Triton at room temperature for 10 min. Cells were blocked with Super Block (ScyTek) at room temperature for 1 hour. Primary antibodies (see Supplementary information) were diluted in blocking buffer and incubated at 4 °C overnight. Alexa Fluor secondary antibodies (Molecular Probes) were diluted in blocking buffer and incubated at room temperature for 1 hr. DAPI (Molecular Probes) was used to stain the nuclei with incubation at room temperature for 10 min. The immunofluorescence was visualized using 63X oil objective with ZEISS Axio Observer Z1 microscope. The criteria for the SK-N-SH cells positive for co-localization with TIAR or TDP-43 (Fig. 5c,d) were defined as the cells containing more than three cytoplasmic RBM45 granules that co-localized with TIAR or TDP-43. The total number of transfected cells (cell numbers > 50 per experiment) and the cells with positive co-localization were counted. The percentage of the SK-N-SH cells positive for co-localization with TIAR or TDP-43 were calculated as [(number of cells positive for co-localization)/(total number of transfected cells)] × 100%. Statistical analysis was performed using two-tailed paired t-test from three experiments. For EGFP-RBM45 live cell imaging, the cells were grown on 35 mm glass-bottom dish and visualized using the 63X oil objective with ZEISS Axio Observer. Images were deconvolved using a maximum likelihood algorithm with the Huygens Deconvolution software (SVI)^47^.

### Proximity ligation assay

Cells were fixed, permeabilized, and blocked as described above. A pair of primary antibodies (see Supplementary information) produced in rabbit and mouse was used for each PLA assay. Primary antibodies were diluted in blocking buffer and incubated at 4 °C overnight. Cells probed with single antibody (Fig. 4d, panel 2 and 3) were also supplemented with IgG antibodies raised in the opposite species. The PLA probe incubation, ligation and amplification processes were performed according to the manufacturer’s instructions (Duolink, Sigma). The PLA signals were visualized using the 63X oil objective with ZEISS Axio Observer Z1.

### Immunoblot

Protein samples were mixed with 4× SDS loading buffer and heated at 90 °C for 7 min, resolved on the Bolt 4–12% Bis-Tris Plus Gel (Life Technologies) or 3–8% Tris-Acetate Gel (Life Technologies), and transferred to Immobilon-FL PVDF membrane (Millipore). The membranes were blocked with Odyssey Blocking Buffer (LiCOR) for 1 hr. The antibodies (see Supplementary information) were diluted in Odyssey Blocking Buffer with 0.1% Tween-20. Primary antibody incubation was performed at room temperature for 1 hr or 4 °C overnight. The IRDye-conjugated secondary antibody (LiCOR) incubation was performed at room temperature for 1 hr. The membranes were scanned using the Odyssey CLx Infrared Imaging System (LiCOR) and quantification was performed using Image Studio software Ver 3.1 (LiCOR). Specificity of each RBM45 antibody was demonstrated using siRNA knockdown of RBM45 expression, as shown in Figure S1c (Supplementary Information).

### DSS in-cell crosslinking

Disuccinimidyl suberate (DSS) in-cell crosslinking was performed as previously described^28^,^29^. The DSS (Thermo Scientific) stock solution was prepared in DMSO immediately before use. Freshly harvested cells were washed with PBS twice and cell pellets resuspended in 10X volume of PBS containing 1 mM DSS. Samples were mixed by gentle agitation at room temperature for 10 min. The crosslinking reaction was quenched by the addition of 1 M Tris (pH7.5) to 50 mM final concentration and mixed by gentle agitation at room temperature for 15 min. Cells were lysed with RIPA buffer and sonicated at 4 °C in a water bath sonicator (Misonix Sonicator 3000) at level 2 for 4 cycles (15 sec on/30 sec off). The sonicated lysate was cleared by centrifugation at 16,000 × g at 4 °C for 10 min and the supernatant was collected for analysis.

### Formaldehyde in-cell crosslinking

Formaldehyde in-cell crosslinking was performed prior to immunoprecipitation. Formaldehyde is a mild and reversible crosslinker with very short spacer length (2.3–2.7 Å) and cross-links only closely associated proteins^48^. Cells were suspended in PBS containing 0.1% formaldehyde and incubated at room temperature for 7 min with gentle agitation. The suspension was spun for 3 min at 1800 × g at room temperature and the supernatant was discarded. The pellet was washed with cold 1.25 M glycine in PBS twice to quench the crosslinking reaction. The pellet was further washed in PBS and lysed with NP40 lysis buffer (50 mM HEPES pH 7.6, 150 mM KCl, 2 mM EDTA, 0.5% NP-40, 0.5 mM DTT and protease/phosphatase inhibitors) at 4 °C and sonicated at with water bath sonicator (Misonix Sonicator 3000) at level 2 for 4 cycles (15 sec on/30 sec off). The sonicated lysate was cleared by centrifugation at 16,000 × g at 4 °C for 10 min and the supernatant was saved for further analysis.

### Immunoprecipitation

Cells were lysed with NP40 lysis buffer (50 mM HEPES pH 7.6, 150 mM KCl, 2 mM EDTA, 0.5% NP40, 0.5 mM DTT and protease/phosphatase inhibitors) at 4 °C for 15 min. The lysates were cleared by spinning at maximum speed at 4 °C for 10 min and subsequently used for IP. FLAG-IP was performed using anti-FLAG M2 Affinity Gel (Sigma A2220). IgG-IP control was performed using Mouse IgG-Agarose (Sigma A0919). IP was performed at 4 °C for 2 hr and the beads were washed in NP40 lysis buffer for six times. The proteins were eluted with SDS sample buffer and heated at 95 °C for 20 min to reverse formaldehyde crosslinking. For fractionation-IP, the KCl concentration in the cytoplasmic fraction was adjusted from 60 mM to 430 mM to approximate the salt concentration in the nuclear extract.

### In-cell RNase treatment, crosslinking and immunoprecipitation

Freshly harvested cells were permeabilized with 100 μl PBS containing 0.15% Triton, 3 μl RNase A (Sigma R4642) and proteinase inhibitor for 10 min at room temperature. 400 μl 0.25% formaldehyde/PBS was added to the 100 μl permeabilized mixture so that the final formaldehyde concentration is 0.1%. The subsequent crosslinking and immunoprecipitation were performed as described above.

### Multiple sequence alignment of RBM45

The RBM45 sequences from human (NP_694453.2), chimpanzee (XP_515938.2), bovine (AAI05263.2), mouse (AAH57890.1), rat (NP_695218.1), chicken (NP_001026423.1), lizard (XP_008116425.1), African clawed frog (NP_001080090.1), rainbow trout (CDQ81595.1), zebrafish (NP_001120874.1), sea urchin (XP_785181.2), honey bee (XP_006566988.1), fruit fly (AAF47879.2), and mosquito (XP_315504.4) were aligned with ClustalW algorithm using the BioEdit software.

## Additional Information

**How to cite this article**: Li, Y. *et al.* RBM45 homo-oligomerization mediates association with ALS-linked proteins and stress granules. *Sci. Rep.* **5**, 14262; doi: 10.1038/srep14262 (2015).

## Supplementary Material

Supplementary Information
