# Supplementary material for: RBM45 homo-oligomerization mediates association with ALS-linked proteins and stress granules
Source: Sci Rep. 2015 Sep 22;5:14262. doi: 10.1038/srep14262 (PMC4585734; doi:10.1038/srep14262)

Supplementary information

RBM45 homo-oligomerization mediates association with ALS-linked proteins and stress granules

Yang Li[1], Mahlon Collins[1,2], Rachel Geiser[1], Nadine Bakkar[1], David Riascos[1] and Robert Bowser[1,2,*]

[1]Divisions of Neurology and Neurobiology, Barrow Neurological Institute, St. Joseph's Hospital and Medical Center, Phoenix, Arizona 85013, USA, and [2]University of Pittsburgh School of Medicine, Pittsburgh, Pennsylvania 15261, USA

* Corresponding author. Gregory W. Fulton ALS and Neuromuscular Research Center, Barrow Neurological Institute, Phoenix, Arizona 85013, U.S.A. Tel.: +1 602 406 8989; E-mail: Robert.Bowser@DignityHealth.org

Materials and methods

Antibodies

The primary antibodies used for immunoblot are as follows: rabbit monoclonal RBM45 C-terminal antibody (custom-made, 1:3000), rabbit polyclonal RBM45 antibody 216-265 (Sigma AV41154, 1:3000) (Fig S1). Rabbit polyclonal TDP-43 antibody (Proteintech 10782-2-AP, 1:3000), rabbit polyclonal FUS antibody (Proteintech 11570-1-AP, 1:3000), rabbit monoclonal GAPDH antibody (Cell Signaling 2118S, 1:5000), rabbit monoclonal Lamin A/C antibody (Abcam ab108922, 1:2000), mouse monoclonal β-tubulin antibody (Sigma T4026, 1:5000), rat monoclonal HA antibody (Roche, clone 3F10, 1:3000), and mouse monoclonal FLAG M2 antibody (Sigma F3165, 1:5000). The secondary antibodies used for immunoblot are LiCOR IRDye 800CW or IRDye 680RD antibodies made in goat (1:15000).

The primary antibodies used for immunofluorescence are as follows: rabbit monoclonal RBM45 C-terminal antibody (custom-made, 1:250), mouse monoclonal TDP-43 antibody (Proteintech 60019-2-Ig, 1:250), mouse monoclonal FUS antibody (Proteintech 60160-1-Ig, 1:250), rabbit polyclonal FUS antibody (Proteintech 11570-1-AP, 1:300), mouse monoclonal TIAR antibody (BD Transduction Laboratories 610352, 1:250), mouse monoclonal G3BP antibody (BD Transduction Laboratories 61126, 1:250), rabbit monoclonal HA antibody (Cell signaling 3724S, 1:3000), mouse monoclonal HA antibody (Abcam ab18181, 1:500), and mouse monoclonal FLAG M2 antibody (Sigma F3165, 1:1000). The secondary antibodies used for immunofluorescence are Alexa Fluor 488 or 594 (Molecular Probes) made in goat (1:300).

The primary antibodies used for proximity ligation assay are as follows: mouse monoclonal FLAG M2 antibody (Sigma F3165, 1:2000), rabbit polyclonal TDP-43 antibody (Proteintech 10782-2-AP,

1:1000), mouse monoclonal HA antibody (Abcam ab18181, 1:3000). The PLA probes used were anti-Mouse Plus and anti-Rabbit Minus (Duolink) suggested by manufacturers.

The antibodies used for endogenous protein immunoprecipitation are as follows: rabbit monoclonal RBM45 C-terminal antibody (custom-made), mouse monoclonal TDP-43 antibody (Proteintech 60019-2-Ig). The rabbit monoclonal HA antibody (Cell signaling 3724S) and the mouse monoclonal FLAG M2 antibody (Sigma F3165) were used as controls for immunoprecipitation.

RBM45 knock-down

40% confluent HEK293 cells in 24-well plate were transfected with 15 pmol of the RBM45 ON-TARGETplus siRNA SMARTpool (GE Dharmacon) using the Lipofectamine 2000. Cells were harvested 48 hours post-transfection, lysed and equal amount of total protein were run on immunoblot.

Immunoprecipitation of endogenous proteins

Formaldehyde in-cell crosslinking was performed prior to immunoprecipitation. Cells were lysed with NP40 lysis buffer (50 mM HEPES pH 7.6, 150 mM KCl, 2 mM EDTA, 0.5% NP40, 0.5 mM DTT and protease/phosphatase inhibitors) at 4˚C for 15 min and sonicated at with water bath sonicator (Misonix Sonicator 3000) at level 2 for 4 cycles (15 sec on/30 sec off). The sonicated lysate were cleared by spinning at maximum speed at 4˚C for 10 min and 360 µg total protein was used for IP. Immunoprecipitations were performed using the Pierce Crosslink Magnetic IP/Co-IP Kit (Life technologies) according to the user manual. Briefly, 5 µg of antibodies were pre-conjugated to the Protein A/G magnetic beads. IP was performed at 4˚C for overnight and the beads were washed three times with lysis buffer and one time with H2O. The proteins were eluted with 100 µl low pH elution buffer. The eluates were mixed with SDS sample buffer and heated at 95˚C for 20 min to reverse formaldehyde crosslinking.

Figure legends

Figure S1. Subcellular localization of RBM45.

(**a**) Schematic of RBM45 with the epitope locations of two anti-RBM45 antibodies indicated in blue.

(**b**) Subcellular fractionation and immunoblot showing that endogenous RBM45 is a nuclear protein in human neuroblastoma SHSY5Y, mouse neuroblastoma Neuro2A and HEK293 cells. Equal proportions of nuclear extract and cytoplasmic extract were immunoblotted with RBM45 C-terminal antibody (epitope residues 460-474), TDP-43, GAPDH (cytoplasmic marker) and Lamin A/C (nuclear marker) antibodies. C=Cytoplasmic fraction, N=Nuclear fraction.

(**c**) The specificity of the two RBM45 antibodies in Fig. S1a were confirmed by RBM45 knock-down experiment. HEK293 cells were transfected with either RBM45 siRNA or a control siRNA and lysed 48 hr post-transfection. Equal amount of total proteins were run on immunoblot and detected with either RBM45-E1 internal antibody (epitope 216-265) or RBM45-E2 C-terminal antibody (epitope 460-474). White arrow denotes the RBM45 band detected by the RBM45-E2 antibody. GAPDH or Tubulin antibodies were used as a loading control for the gels. RBM45 siRNA greatly reduced immunoreactivity of the specific band for RBM45.

(**d**) Protein alignment of the NLS region of RBM45 from 14 species. Conserved residues (including homologous residues) are highlighted (conservation threshold=80%). The positively charged residues at both ends of the bipartite NLS (454R, 456K, 469K, 470R, 472R) are highly conserved during evolution.

Figure S2. Homo-oligomerization of RBM45.

(**a**) DSS crosslinked samples from Fig. 3b were resolved on 3-8% Tris-Acetate gel along with a high-molecular-weight size marker (HiMark Protein Standard, Life Technologies) and the LiCOR molecular weight marker. The immunoblot was probed with RBM45 C-terminal antibody. The FLAG-RBM45 monomer, pentamer and the presumed octamer are indicated by arrows. The observed sizes of the monomers and pentamers were indicated next to the bands. The theoretical molecular weights of the monomers and pentamers were indicated below the blot.

(**b**) Whole immunoblots that were probed with TDP-43, β-tubulin and GAPDH antibodies in Fig. 3b.

(**c**) FLAG-RBM45 pentamer is stable with extended time of DSS-crosslinking. Live HEK293 cells expressing FLAG-RBM45 underwent *in vivo* crosslinking using 2 mM DSS for 0-50 minutes. The immunoblot was probed with RBM45 C-terminal antibody. Arrowheads indicate the sizes of FLAG-RBM45 monomer, pentamer and octamer.

(**d**) Homo-oligmerization of endogenous RBM45 from HEK293 and SK-N-SH cells. Live cells were treated with 1mM DSS for 10 minutes. The immunoblot was probed with RBM45 C-terminal antibody.

(**e**) Subcellular fractionation of the DSS crosslinked samples from HEK293 cells expressing FLAG-RBM45 (DSS=1mM). Live cell crosslinking was performed with 1mM DSS for 10 minutes followed by subcellular fractionation. Equal proportions of crosslinked nuclear extract and cytoplasmic extract were immunoblotted with RBM45 C-terminal antibody, TDP-43 (crosslinking positive control), Lamin A/C (nuclear marker) and GAPDH (cytoplasmic marker) antibodies. Oligomers of FLAG-RBM45 were observed in both the nuclear and cytoplasmic fractions, while the dimer form of TDP-43 was observed predominantly in the nucleus.

Figure S3. Interactions between RBM45 and TDP-43 are mediated by the HOA domain.

(**a**) Immunoprecipitation of endogenous proteins in SK-N-SH cells shows that endogenous TDP-43 co-purified with endogenous RBM45. **Left:** endogenous RBM45 was immunoprecipitated with rabbit RBM45 antibody, while rabbit HA antibody was used for IP control. On the immunoblot, TDP-43 was detected with mouse monoclonal Tdp43 antibody (Proteintech 60019-2-Ig), FUS was detected with mouse monoclonal FUS antibody (Proteintech 60160-1-Ig), and RBM45 was detected with rabbit polyclonal RBM45 antibody 216-265 (Sigma AV41154). **Right:** endogenous TDP-43 was immunoprecipitated with mouse TDP-43 antibody, while mouse FLAG antibody was used for IP control. RBM45 was detected with rabbit polyclonal RBM45 antibody 216-265 (Sigma AV41154), and TDP-43 was detected with rabbit polyclonal TDP-43 antibody (Proteintech 10782-2-AP). Endogenous RBM45 appears as the same size as the IgG heavy chain on this immunoblot.

(**b**) The nuclear localization of endogenous TDP-43 is not affected by the overexpression of the cytoplasmic retained RBM45-NLS mutant. Transfection of HA-RBM45 constructs, subcellular fractionation and immunoblot were performed as described in as in Fig. 1d. Endogenous TDP-43 was detected with rabbit TDP-43 antibody (Proteintech 10782-2-AP).

(**c**) RBM45-D4 (Δ286-318) construct exhibits significantly reduced binding to ALS-linked proteins including TDP-43 and FUS when compared to full-length-RBM45. Full-length FLAG-RBM45 or FLAG-D4 construct were expressed in HEK293 cells. FLAG-IP was performed as described previously. Immunoblot analysis shows that both TDP-43 and FUS displayed reduced co-IP% with FLAG-D4 as compared with the full-length FLAG-RBM45. The number on the right displays the percentage of TDP-43 or FUS proteins co-purified with FLAG-D4 as compared with FLAG-FL RBM45.

(**d**) Schematic of HA-RBM45 truncation constructs used in **e**.

(**e**) HA-tagged RBM45 constructs were transfected into the HEK293 cells stably expressing FLAG-TDP-43. FLAG-IP was performed as described previously. The IP fractions were immunoblotted with HA (HA-RBM45 constructs), FLAG (FLAG-TDP-43) and GAPDH (negative control).

Figure S4. Endogenous wild-type FUS is not incorporated into the cytoplasmic stress granules. HA-RBM45 NLS M2 mutant was transfected into SK-N-SH cells, and 48 hr post-transfection, the transfected cells were stressed with 1mM sodium arsenite for 30 minutes followed by immunostaining. The endogenous FUS protein (green) does not co-localize with HA-RBM45 NLS (**a**) or TIAR (**b**).

FUS was immunostained with rabbit polyclonal FUS antibody (green), HA-RBM45 NLS was immunostained with mouse anti-HA antibody (red), and TIAR was stained with mouse monoclonal anti-TIAR antibody (red). Nuclei were labeled with DAPI. Scale bar: 10 μm.

Figure S1

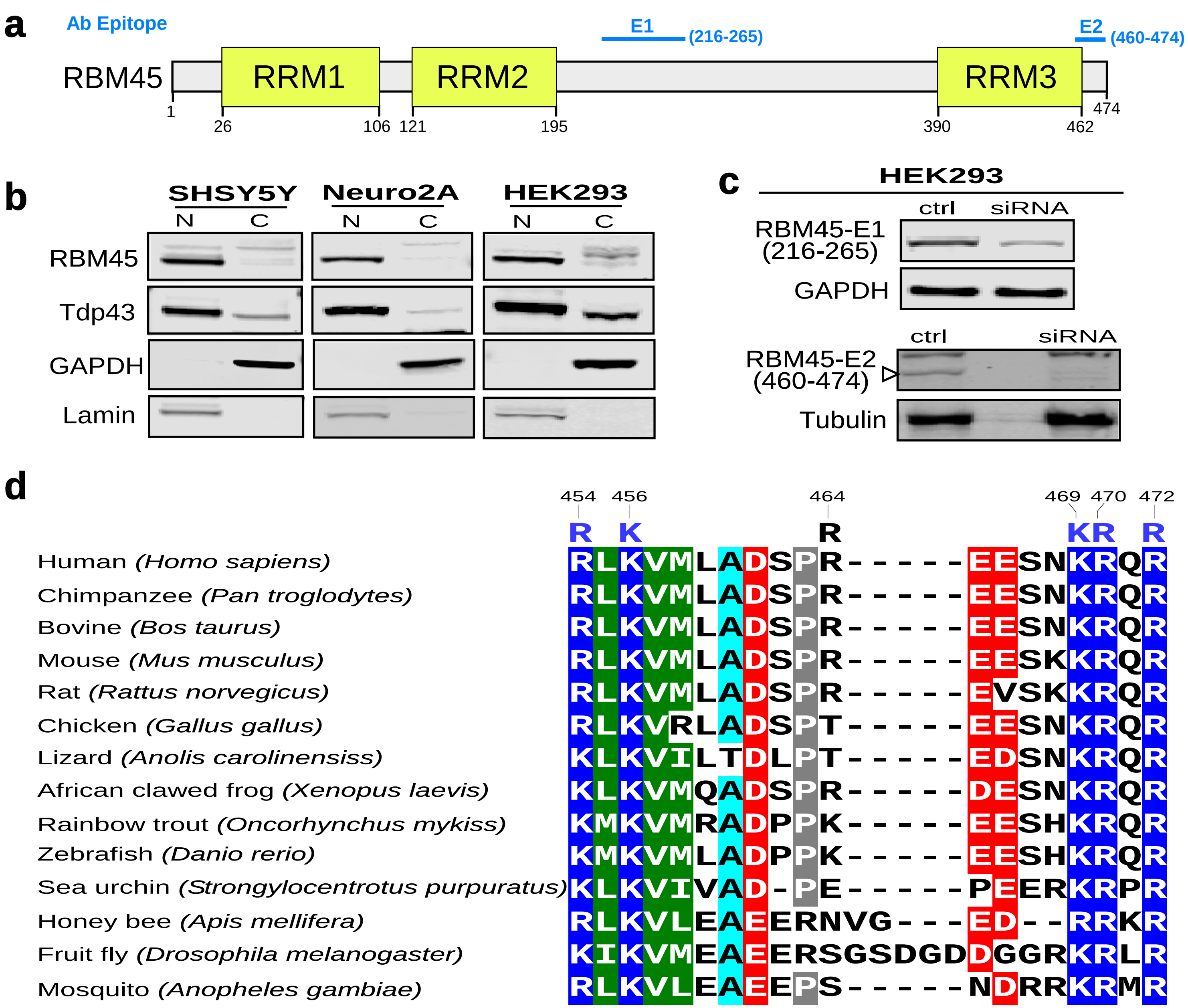
a
Ab Epitope
E1
(216-265)
E2
(460-474)
RBM45
RRM1
RRM2
RRM3
1
26
106
121
195
390
462
474
b
SHSY5Y
Neuro2A
HEK293
N
C
RBM45
Tdp43
GAPDH
Lamin
c
HEK293
ctrl
siRNA
RBM45-E1
(216-265)
GAPDH
RBM45-E2
(460-474)
Tubulin
d
454
456
464
469
470
472
R K R KR R
Human (Homo sapiens) RLKVMLADSPR------EESNKRQR
Chimpanzee (Pan troglodytes) RLKVMLADSPR------EESNKRQR
Bovine (Bos taurus) RLKVMLADSPR------EESNKRQR
Mouse (Mus musculus) RLKVMLADSPR------EESKKRQR
Rat (Rattus norvegicus) RLKVMLADSPR------EVSKKRQR
Chicken (Gallus gallus) RLKVRLADSPT------EESNKRQR
Lizard (Anolis carolinensiss) KLKVILTDLPT------EDSNKRQR
African clawed frog (Xenopus laevis) KLKVMQADSPR------DESNKRQR
Rainbow trout (Oncorhynchus mykiss) KMKVMRADPPK------EESHKRQR
Zebrafish (Danio rerio) KMKVMLADPPK------EESHKRQR
Sea urchin (Strongylocentrotus purpuratus) KLKVIVAD-PE------PEERKRPR
Honey bee (Apis mellifera) RLKVLEAEERNVG---ED--RRKR
Fruit fly (Drosophila melanogaster) KIKVMEAEERSGSDGDDGGRKRLR
Mosquito (Anopheles gambiae) RLKVLEAEEPS------NDRRKRMR

Figure S2

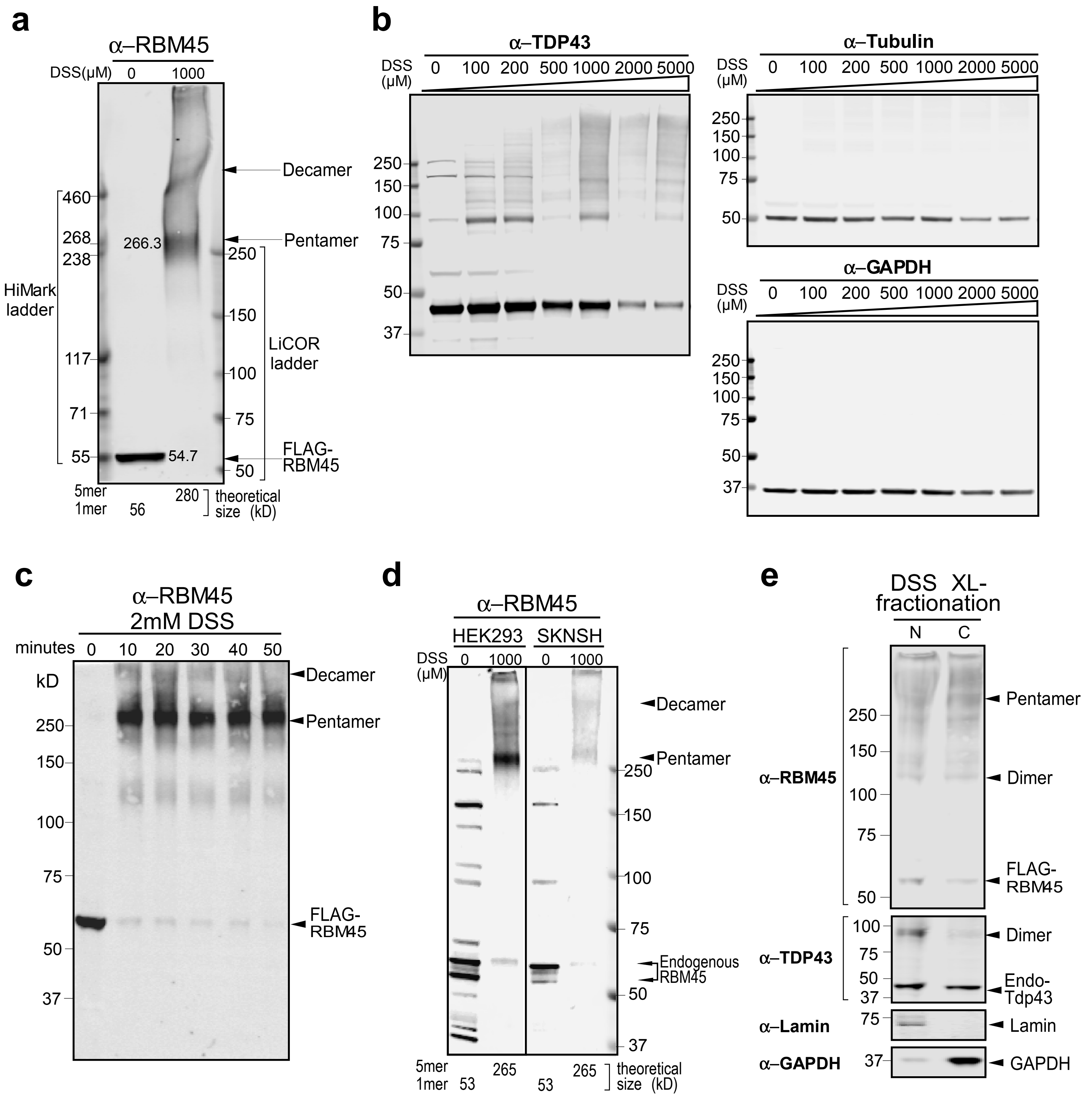
a
α–RBM45
DSS(μM) 0 1000
Decamer
Pentamer
FLAG-RBM45
460
268
238
117
71
55
266.3
54.7
250
150
100
75
50
HiMark ladder
LiCOR ladder
5mer 280
1mer 56
theoretical size (kD)
b
α–TDP43
DSS (μM) 0 100 200 500 1000 2000 5000
250
150
100
75
50
37
α–Tubulin
DSS (μM) 0 100 200 500 1000 2000 5000
250
150
100
75
50
α–GAPDH
DSS (μM) 0 100 200 500 1000 2000 5000
250
150
100
75
50
37
c
α–RBM45
2mM DSS
minutes 0 10 20 30 40 50
kD
250
150
100
75
50
37
Decamer
Pentamer
FLAG-RBM45
d
α–RBM45
HEK293 SKNSH
DSS (μM) 0 1000 0 1000
Decamer
Pentamer
250
150
100
75
50
37
Endogenous RBM45
5mer 265 265
1mer 53 53
theoretical size (kD)
e
DSS XL-fractionation
N C
α–RBM45
250
150
100
75
50
Pentamer
Dimer
FLAG-RBM45
α–TDP43
100
75
50
37
Dimer
Endo-Tdp43
α–Lamin
75
Lamin
α–GAPDH
37
GAPDH

Figure S3

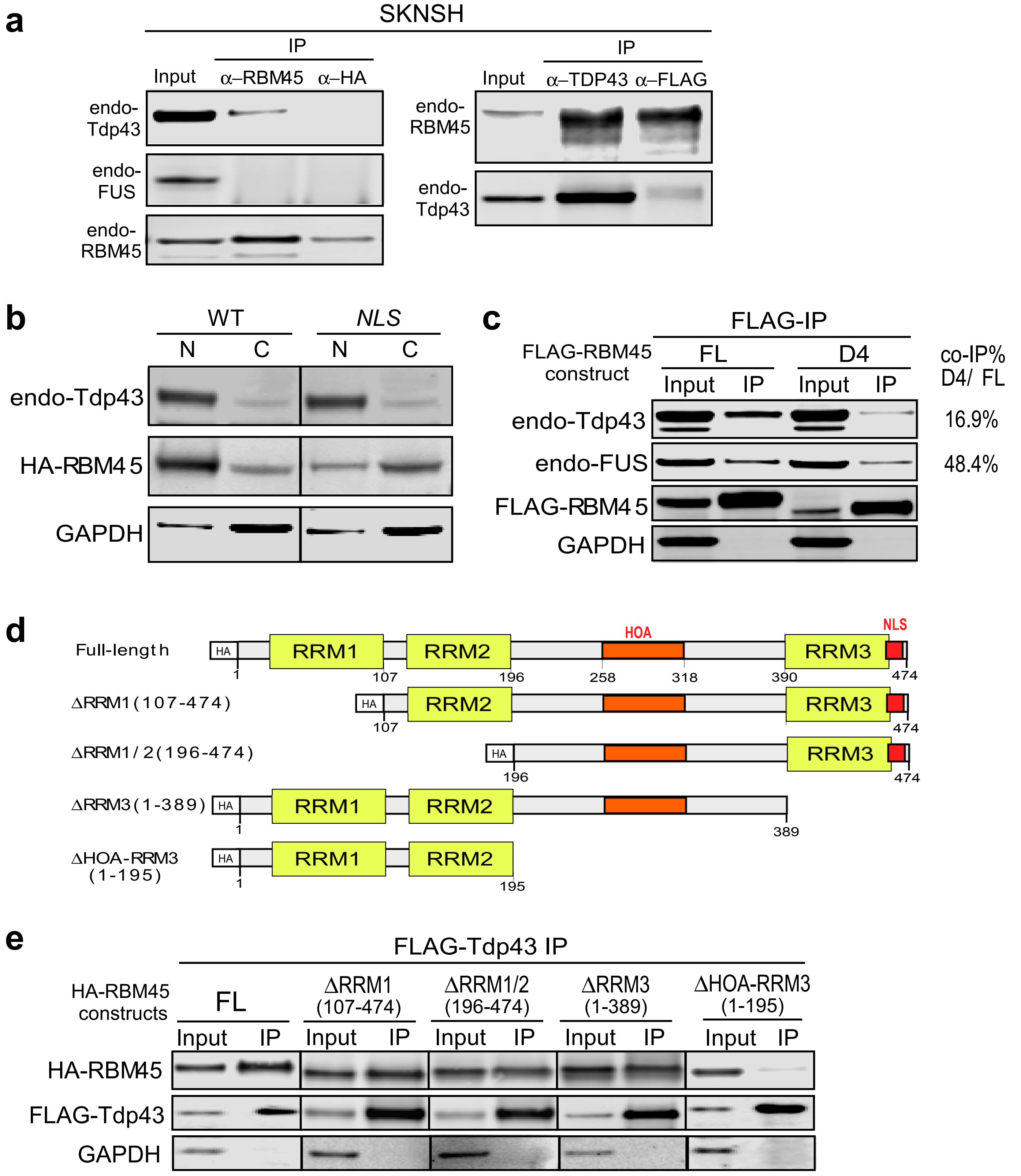

a
SKNSH
IP
Input
α–RBM45
α–HA
endo-Tdp43
endo-FUS
endo-RBM45
IP
Input
α–TDP43
α–FLAG
endo-RBM45
endo-Tdp43
b
WT
NLS
N
C
N
C
endo-Tdp43
HA-RBM45
GAPDH
c
FLAG-IP
FLAG-RBM45 construct
FL
D4
Input
IP
Input
IP
co-IP% D4/ FL
endo-Tdp43
16.9%
endo-FUS
48.4%
FLAG-RBM45
GAPDH
d
Full-length
HA
RRM1
RRM2
HOA
RRM3
NLS
1
107
196
258
318
390
474
ΔRRM1(107-474)
ΔRRM1/2(196-474)
ΔRRM3(1-389)
389
ΔHOA-RRM3 (1-195)
195
e
FLAG-Tdp43 IP
HA-RBM45 constructs
FL
ΔRRM1 (107-474)
ΔRRM1/2 (196-474)
ΔRRM3 (1-389)
ΔHOA-RRM3 (1-195)
Input
IP
HA-RBM45
FLAG-Tdp43
GAPDH

Figure S4

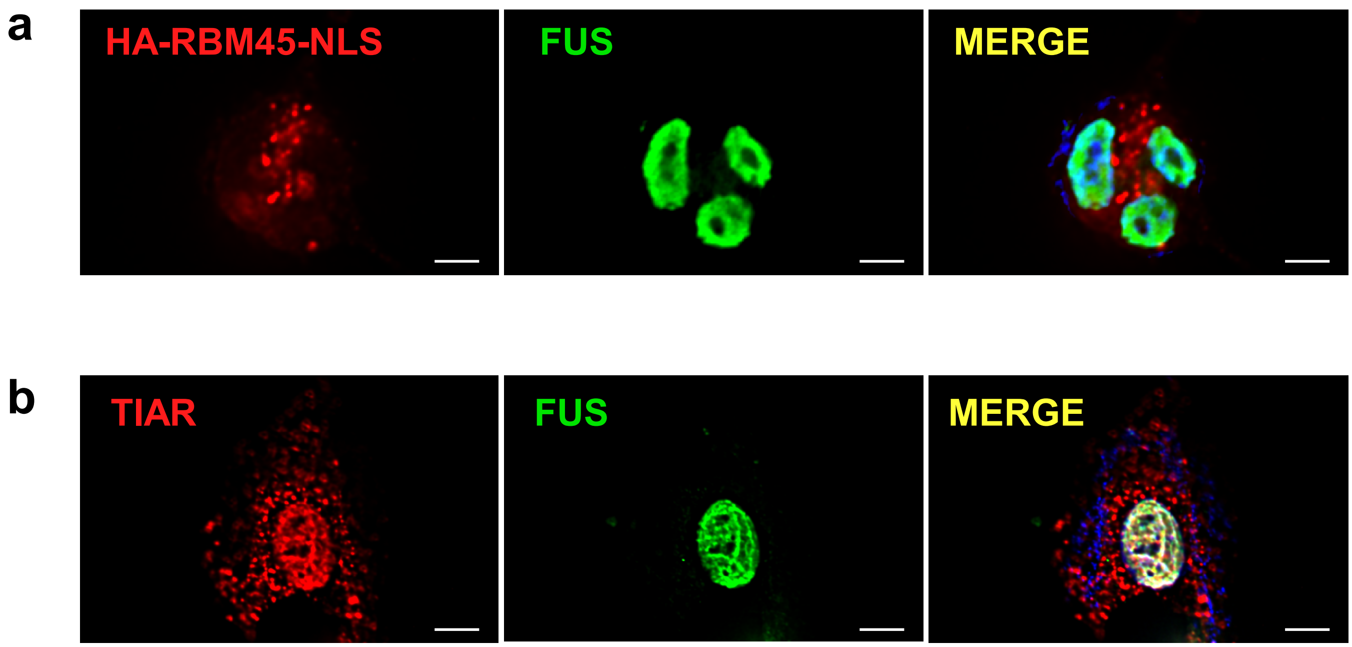

Supplement: Supplementary Information [file srep14262-s1.pdf]
